# Autoantibody Profiling in Lupus Patients using Synthetic Nucleic Acids

**DOI:** 10.1038/s41598-018-23910-5

**Published:** 2018-04-03

**Authors:** Martin Klecka, Christina Thybo, Claudia Macaubas, Ilia Solov’yov, Julia Simard, Imelda Maria Balboni, Emily Fox, Anne Voss, Elizabeth D. Mellins, Kira Astakhova

**Affiliations:** 1 Department of Chemistry, Technical University of Denmark, Kemitorvet 206, 2800 Kgs Lyngby, Denmark; 2 Department of Physics, Chemistry and Pharmacy, University of Southern Denmark, Campusvej 55, 5230 Odense M, Denmark; 3 Department of Pediatrics, Program in Immunology, Stanford University School of Medicine, 269 Campus Drive, Stanford, California, 94305 USA; 4 Department of Health and Research Policy, Stanford University School of Medicine, 150 Governor’s Lane, Stanford, California, 94305 USA; 5 Department of Pediatrics, Division of Allergy, Immunology, and Rheumatology, Stanford University, 700 Welch Rd. Suite 301, Stanford, California, 94304 USA; 6 Department of Rheumatology, Odense University Hospital, J. B. Winsløws Vej 19, 2, 5000 Odense C, Denmark

## Abstract

Autoantibodies to nuclear components of cells (antinuclear antibodies, ANA), including DNA (a-DNA), are widely used in the diagnosis and subtyping of certain autoimmune diseases, including systemic lupus erythematosus (SLE). Despite clinical use over decades, precise, reproducible measurement of a-DNA titers remains difficult, likely due to the substantial sequence and length heterogeneity of DNA purified from natural sources. We designed and tested a panel of synthetic nucleic acid molecules composed of native deoxyribonucleotide units to measure a-DNA. ELISA assays using these antigens show specificity and reproducibility. Applying the ELISA tests to serological studies of pediatric and adult SLE, we identified novel clinical correlations. We also observed preferential recognition of a specific synthetic antigen by antibodies in SLE sera. We determined the probable basis for this finding using computational analyses, providing valuable structural information for future development of DNA antigens. Synthetic nucleic acid molecules offer the opportunity to standardize assays and to dissect antibody-antigen interactions.

## Introduction

Autoantibodies to nuclear components of the cell (antinuclear antibodies, ANA) are detected in patients with a variety of autoimmune diseases (reviewed in^1^). Among ANA, antibodies to double stranded DNA (a-dsDNA) are particularly characteristic of SLE, a multisystem inflammatory autoimmune disease with diverse clinical and serological manifestations and unknown etiology^2^. Older healthy individuals can have increased a-dsDNA titers without any symptoms of autoimmune disease^3^. However, in the context of SLE, immune complexes with these antibodies typically fix complement and cause acute and chronic blood vessel and tissue inflammation and damage^4^. Anti-DNA antibodies can cross-react with NMDA (N-methyl-D-aspartate) receptors of the brain and cause central nervous system pathology^5^. In addition, anti-DNA/DNA complexes stimulate mononuclear cell release of pro-inflammatory cytokines (e.g., IL-1β, IL-8 and TNFα) and IL-10, which may polarize the immune reaction towards the T helper 2 (Th2) pathway and support more autoantibody production^6^.

In most patients with SLE, the disease course is characterized by flares and remissions^7^. Early detection and treatment of flares in SLE may improve short-term outcomes and reduce morbidity over the long-term^8^. Antibodies to dsDNA and to Smith antigen, a non-histone nuclear protein composed of several polypeptides, have validated diagnostic value in SLE, and increased anti-ds DNA titers are associated with disease flare in some patients, but not universally^9^. Finding additional biomarkers of SLE activity is the goal of many current studies, with some recent candidates being cell-bound complement-activated proteins C4d and C3d, several urinary proteins, such as transferrin, CC-chemokine ligands and hepcidins, RNA, microRNA, and epigenetic profiles of circulating immune cells, (as reviewed in Liu *et al*., ref.^10^). However, convincing data on the value of ANA, such as a-dsDNA, detected by enzyme-linked immunosorbent assay (ELISA) as a biomarker of disease are lacking.

The common sources of DNA antigens for detection of ANA include calf thymus DNA (CTD), PCR amplicons of different length, and plasmid DNA, which are highly heterogeneous and are used in ANA detection without knowledge of DNA sequence. Using CTD, accurate detection of a-single-stranded (ss) DNA versus a-dsDNA is challenging, because CTD is a mixture of ss- and ds-DNA with a high proportion (~90%) of dsDNA^11,12^. In addition, even highly pure CTD contains covalently bound phosphopeptides that might influence antibody binding. Alternatively, *Crithidia luciliae*, a flagellate protist with a kinetoplast rich in dsDNA, can be used as antigen^9^. Although Crithidia DNA has a higher purity than CTD, the detection of a-DNAs with this substrate is not sequence specific.

Structural information on interaction of a-DNA with corresponding antigens, though limited^13–16^, suggests sequence specific interaction with defined nucleotides^17^. Current clinical tests do not take this into account^9^. The use of natural antigens likely contributes to inconsistency in results between different laboratories and may hamper correlations with clinical parameters^18,19^. Using pure, sequence-controlled DNA would enable more consistent detection, discrimination, and possible subtyping of a-DNAs. Information from a-DNAs with known sequence specificity would help provide a strong theoretical basis for antibody-DNA recognition. Moreover, structural data on antibody-DNA complexes could be used in the design of antigens with improved specificity, which is of crucial importance to clinical diagnostics^18,19^. One successful example includes G-quadruplex DNA, which allowed subtyping of SLE patients and showed correlation of a-DNA titers with disease activity^20^. Synthetic antigens could allow establishment of previously unachievable standardization of the a-DNA assays and might open up the exciting possibility of treatment by specific binding and clearance of reactive a-DNAs^21^.

We have shown the unique specificity and sensitivity of synthetic DNA oligonucleotides containing locked nucleic acids (LNA) for recognition by monoclonal a-dsDNAs^22^. Recently, other investigators explored rationally designed peptoid antigens for SLE diagnostics^23^. Here, we report a series of new synthetic DNA antigens and demonstrate their applicability for detection of corresponding antibodies by ELISA in patients with pediatric onset SLE (pSLE) or adult-onset SLE. Our studies confirm high binding affinity of the new antigens compared to natural DNA. We find mixed a-ssDNA/a-dsDNA profiles that vary between patients. Increased antibody titers to synthetic dsDNA correlate with high disease activity, measured by SLEDAI. We show that levels of autoantibodies to particular synthetic nucleic acid antigens in SLE differ among adults and children. The a-dsDNA profiles in SLE also differ from those in patients with another autoimmune disease, ANA-positive polyarticular juvenile idiopathic arthritis, indicating specificity. In addition, using computational methods, we identify specific interactions between dsDNA and corresponding antibodies.

## Results

**Figure 1:**
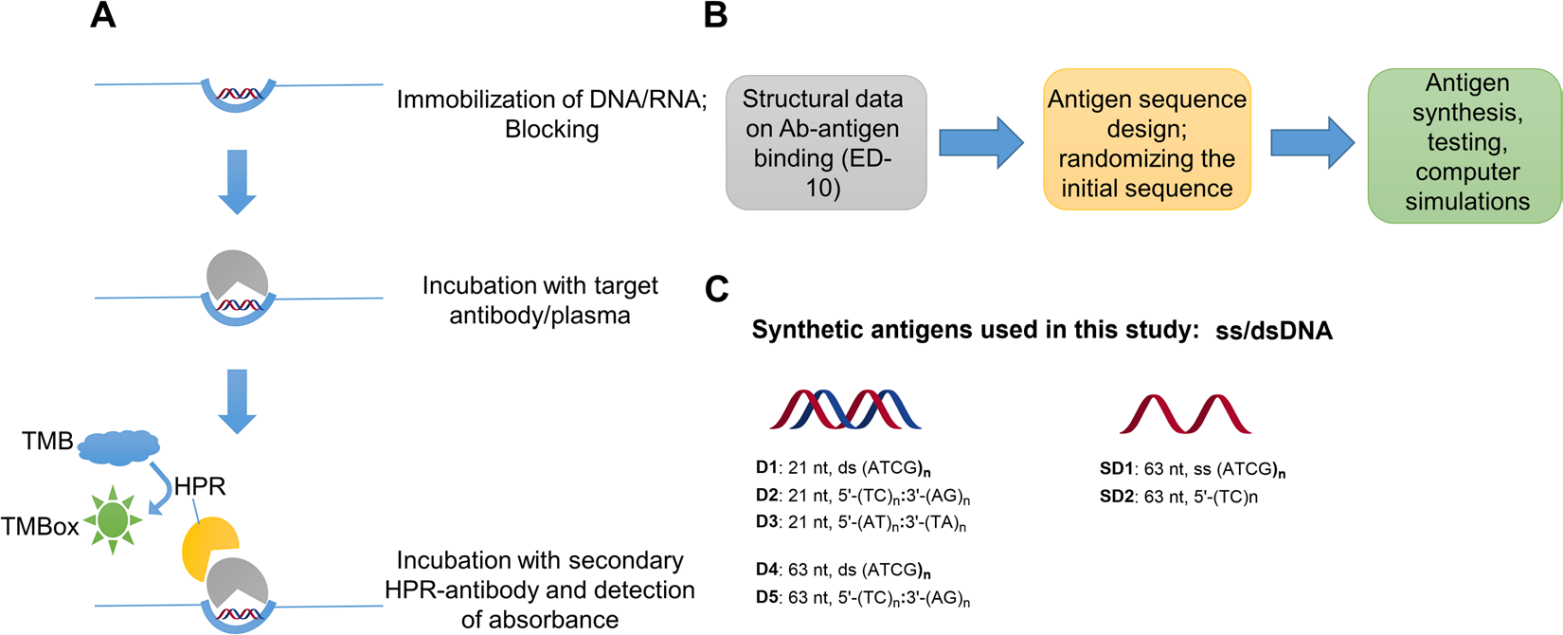
General scheme of ELISA assay and sequences of applied antigens. (**A**) ELISA assay: Step 1. Immobilization of antigen and blocking; Step 2. Incubation with monoclonal antibody or plasma sample; Step 3. Incubation with secondary HPR-conjugated antibody (anti-IgG or anti-IgM); Step 4. Incubation with substrate for color generation; measurement of absorbance. (**B**) General approach for the antigen development applied in this study. (**C**) Synthetic nucleic acid antigens used in this study. For the sequences of antigens, see Methods.

The major goal for this study was to develop a sensitive, specific and reproducible test for a-DNA in human samples. For measuring the amount of a-DNA IgG and IgM, we selected the a-DNA ELISA. ELISA is a straightforward and well-established assay that allowed us to study the effect of DNA sequence on binding of polyclonal antibodies in a time and cost- effective way^1^. Microtiter plates for ELISA were coated with the nucleic acid antigen of choice (see below). After washing, secondary antibodies, specific for human IgG or IgM and conjugated with peroxidase were added. After washing again to remove unbound detection antibodies, TMB substrate was added and the extent of the colorometric reaction was measured and compared among different antigens as a proxy for the amount of bound anti-nucleic acid antibodies; see Fig. 1A. Details on the assay are given in Supplementary information, chapter S1.

For the ELISA antigens, we used a panel of synthetic DNA molecules, including a set of double stranded DNA (D1-D5) and a set of single stranded DNA (SD1, SD2). The design of the synthetic DNA antigens was based on previous data for DNA-antibody binding^13–17^ and additional molecular modelling of 40 DNA sequences (22; Fig. 1B,C). As a control antigen, we used calf thymus DNA (CTD).

To test the a-DNA ELISA in clinically relevant samples, we collected blood from children newly diagnosed with pSLE (n = 27), SLE positive adults (n = 244), healthy controls (n = 60) and ANA-positive polyJIA patients (n = 14) with on-going disease^24,25^. Demographic and clinical information on the patients are shown in Supplementary Tables S1–S4 (Suppl. Section S2).

We ensured that antibody binding to the antigens reached the binding equilibrium under the applied incubation conditions (1.5 h, 37 °C); (Supplementary Fig. S3 in Suppl. Section S1). Cutoff values for weak positive and positive signals were determined separately for each antigen by an arbitrary statistical method, i.e. as a 2- and 3-fold increase respectively of A_450_ above the mean value for the healthy controls^26^.

**Figure 2:**
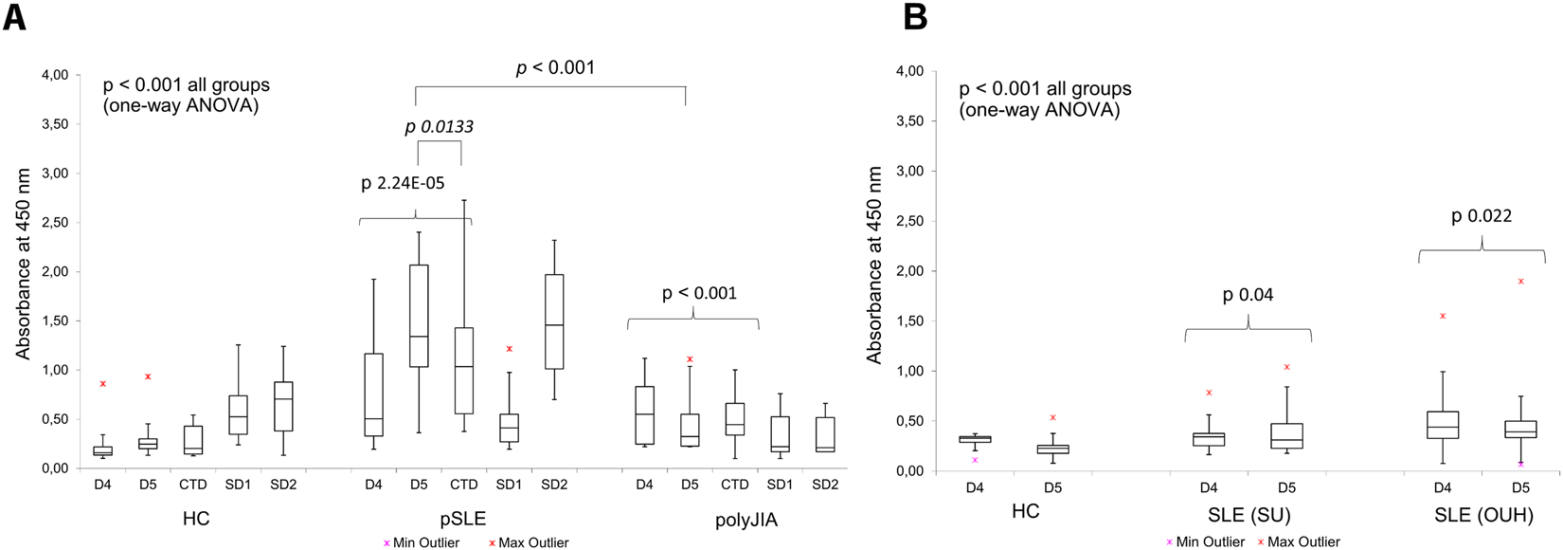
ELISA tests of patient samples grouped according to diagnosis. Absorbance values were corrected to total plasma protein determined by Bradford assay, see Methods. P value > 0.05 was considered statistically significant.

A striking finding and the most important result of this work was the preferential binding of polyclonal a-DNA antibodies from SLE samples to the antigen D5 compared to both D4 and CTD (Fig. 2; sequence of **D5**: (5′-TCCTCTCTTTCTCTTTCTCTTTCCTCTCTTTCTCTTTCTCTTTCCTCTCTTTCTCTTTCTCTT-3′): (5′-AAGAGAAAGAGAAAGAGAGGAAAGAGAAAGAGAAAGAGAGGAAAGAGAAAGAGAAAGAGAGGA-3′)). High titers of a-D5 antibodies were observed in 19 pSLE samples (70%), including 9 (33%) that were negative for CTD. For 89% of a-D5 positive pSLE samples, reactivity towards D5 was two-fold higher than for D4 and up to 10-fold higher than D5-reactivity of JIA samples, indicating both the antigenic specificity and disease specificity of the reactivity. Two-tailed t-test assuming unequal variances confirmed statistically significant differences between D5 titers for pSLE and polyJIA groups (p = 4.9×10^−9^; F 5.93 > F critical 2.065). When single-stranded DNA antigens, SD1 and SD2, were used, pSLE samples showed higher titers of a-ssDNA toward antigen SD2, compared to JIA or healthy control samples (p < 0.001). Thus, greater binding of pSLE antibodies was observed for both ds and ss synthetic DNA targets (Fig. 2). In contrast to pSLE, fewer adults had elevated levels of a-D4 (7.3% vs. 26% for pSLE), and of a-D5 (19% (OUH) and 23% (SU) vs. 70% for pSLE). a-D5 levels in adults did not correlate with a-D4, clinical a-dsDNA or a-CTD (Supplementary Tables S5,S6 in Suppl. section S3).

Dose-response curves for new antigens and controls indicated saturation values for antibodies in most pSLE samples binding to D5, CTD and in some cases, D4; three samples appeared to have lower a-D4 titers and did not saturate (Supplementary Figs S5 and S6, Suppl. Section S4). In addition, the hierarchy of sample binding to the synthetic antigens varied. For example, sample pSLE6 has the lowest titers against D4 but not against D5 or CTD. Sample pSLE20 has the lowest titers of a-D5 with high titers of D4 and CTD relative to the other samples.

DNA antigens D1-D3 allowed us to further investigate the sequence specificity of a-DNAs. As shown in Supplementary Fig. S6, antibodies in pSLE samples had higher binding signals to sequence D2 than to D3 and D1. This suggests the importance of interactions between certain dinucleotides and antibodies and might indicate a primary immunogenic role of particular nucleotide sequences in the development of the a-DNA autoantibody response/population^27^.

**Figure 3:**
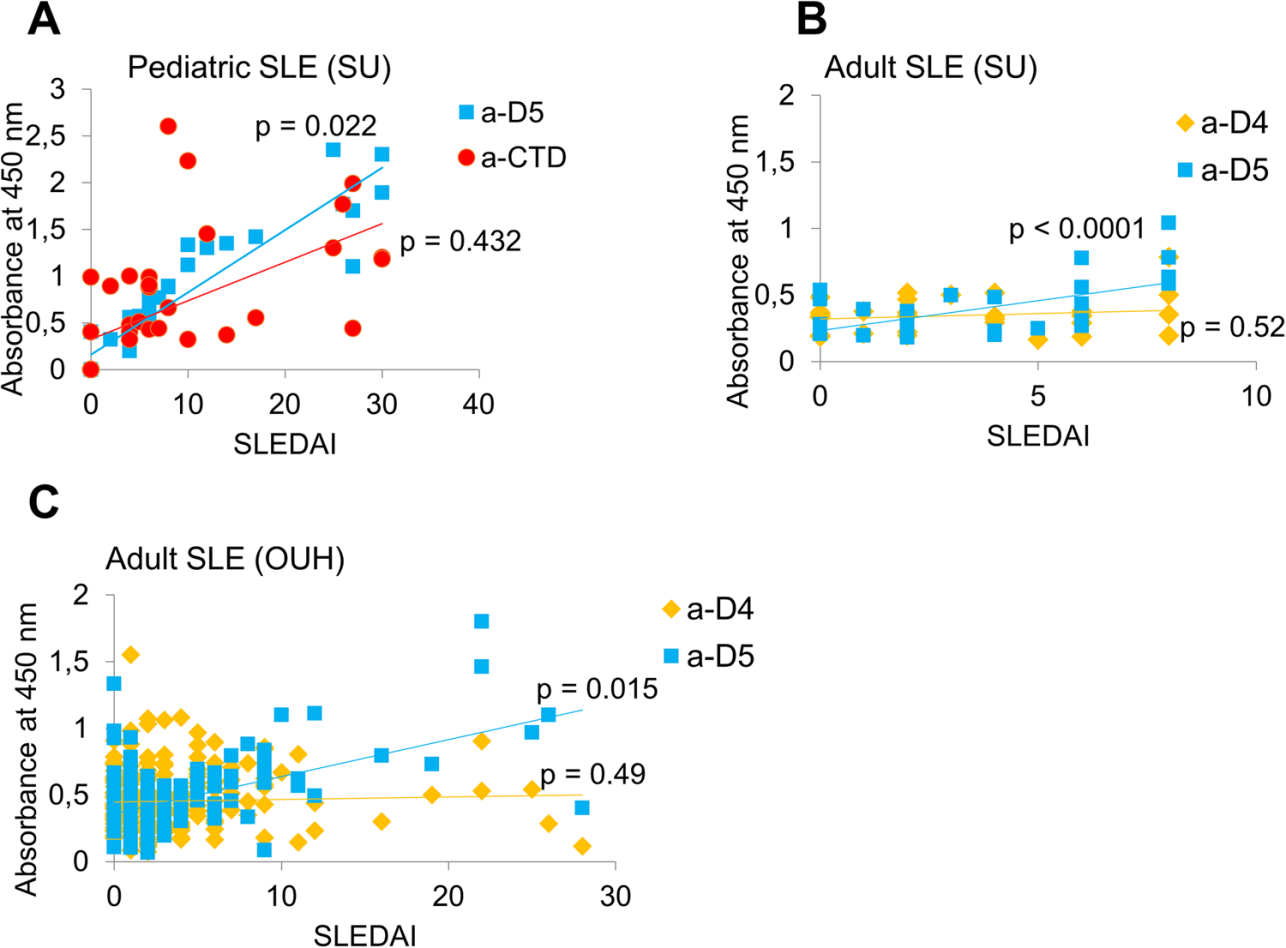
Correlations between disease activity at onset (SLEDAI) and antibody titers. Correlations were determined using OLS for independent groups, as described in Supplementary Information. (**A**) pSLE cohort; (**B**) adult SLE subjects from SU; (**C**) adult SLE cohort from OUH. SU = Stanford University. OUH = Odense University Hospital.

To explore potential clinical application for a-D5 antibodies, we assessed correlations with clinical parameters, including disease flares and medications, using multi-parameter ordinary least squares (OLS) in R^28^, as described in Supplementary Information, Suppl. Section S3. According to OLS test, the a-D5 titers correlated with SLE disease activity index (SLEDAI) in pediatric and adult samples (p = 0.022, 0.0008 (SU) and 1.6 × 10^−11^ (OUH)). In contrast, for the same patients, there was no statistically significant correlation between anti-CTD, a-D4 antibodies and SLEDAI scores (p = 0.432; Fig. 3). Interestingly, 7 of 27 (26%) pSLE patients had elevated a-D4 titers. For 6 pSLE patients, a-D4 reactivity correlated with SLEDAI and anti-phospholipid antibodies, but not other clinical parameters measured (e.g. anti-Smith or anti-ribonucleoprotein (Anti-U1RNP) antibodies; see Supplementary Table S5). Neither a-D4 nor a-D5 levels correlated with medication use. None of SLE subjects was treated with medications associated with drug-induced SLE, which also argues that a-DNA were specific to the disease and not to treatment^29^.

**Figure 4:**
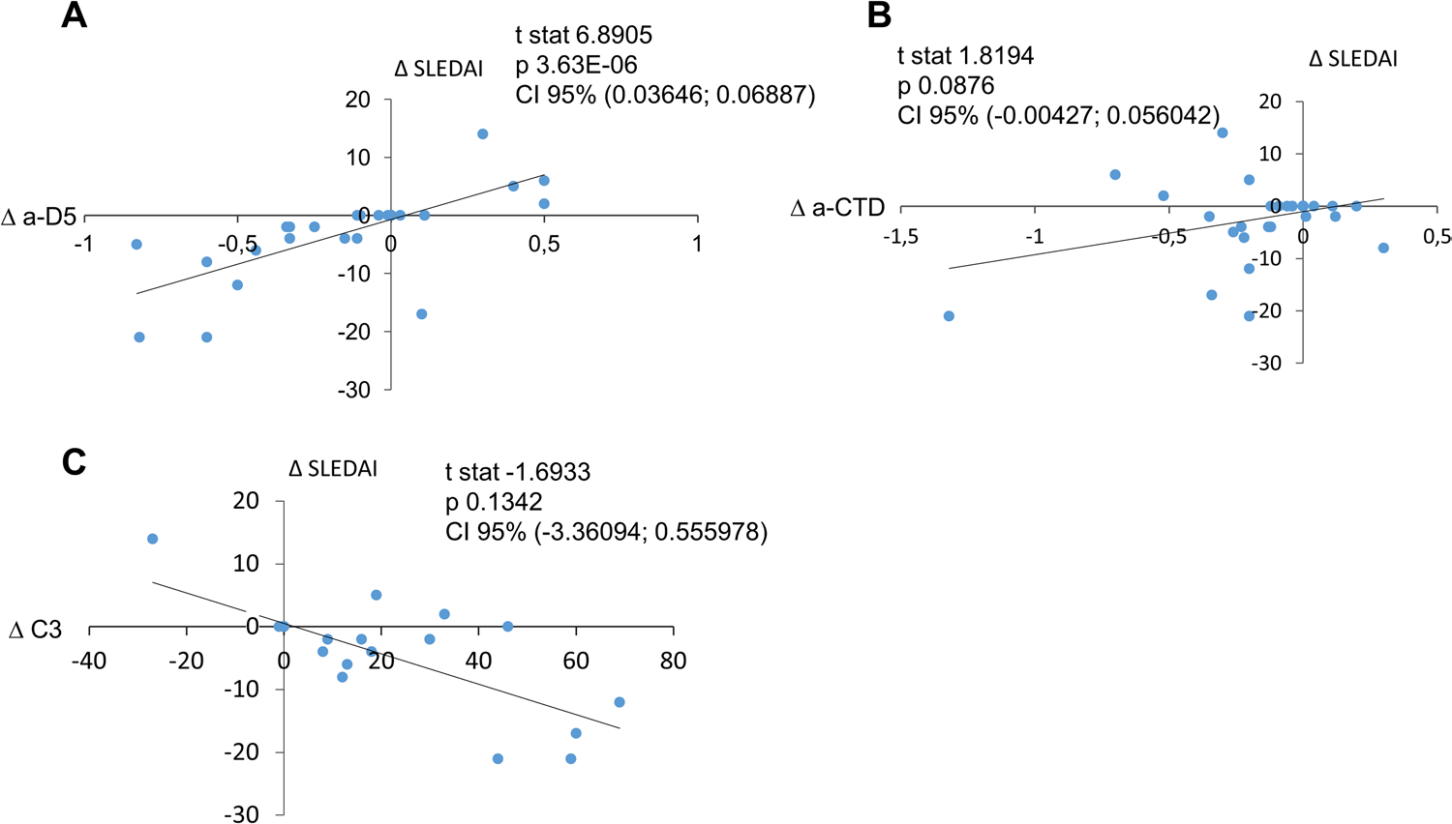
Results of longitudinal IgG ELISA assay for pSLE patients. Changes in disease activity versus changes of corresponding laboratory parameters over time (longitudinal assay). All results are based on a total of 32 visits for 8 patients. Delta values were calculated by subtracting values of a defined parameter at each visit from the value from the previous visit. Resulting plots were analyzed in R.

Next, we tested for correlations between autoantibody titers and clinical parameters for selected patients during the period from disease onset to 64 months of treatment; see Supplementary Information, Section S5). Changes in a-dsDNA titers correlated positively with increased SLEDAI, whereas the predictive value of changes in a-CTD titers and complement C3 were low (Fig. 4 and Supplementary Table S8, Suppl. Section S5).

**Figure 5:**
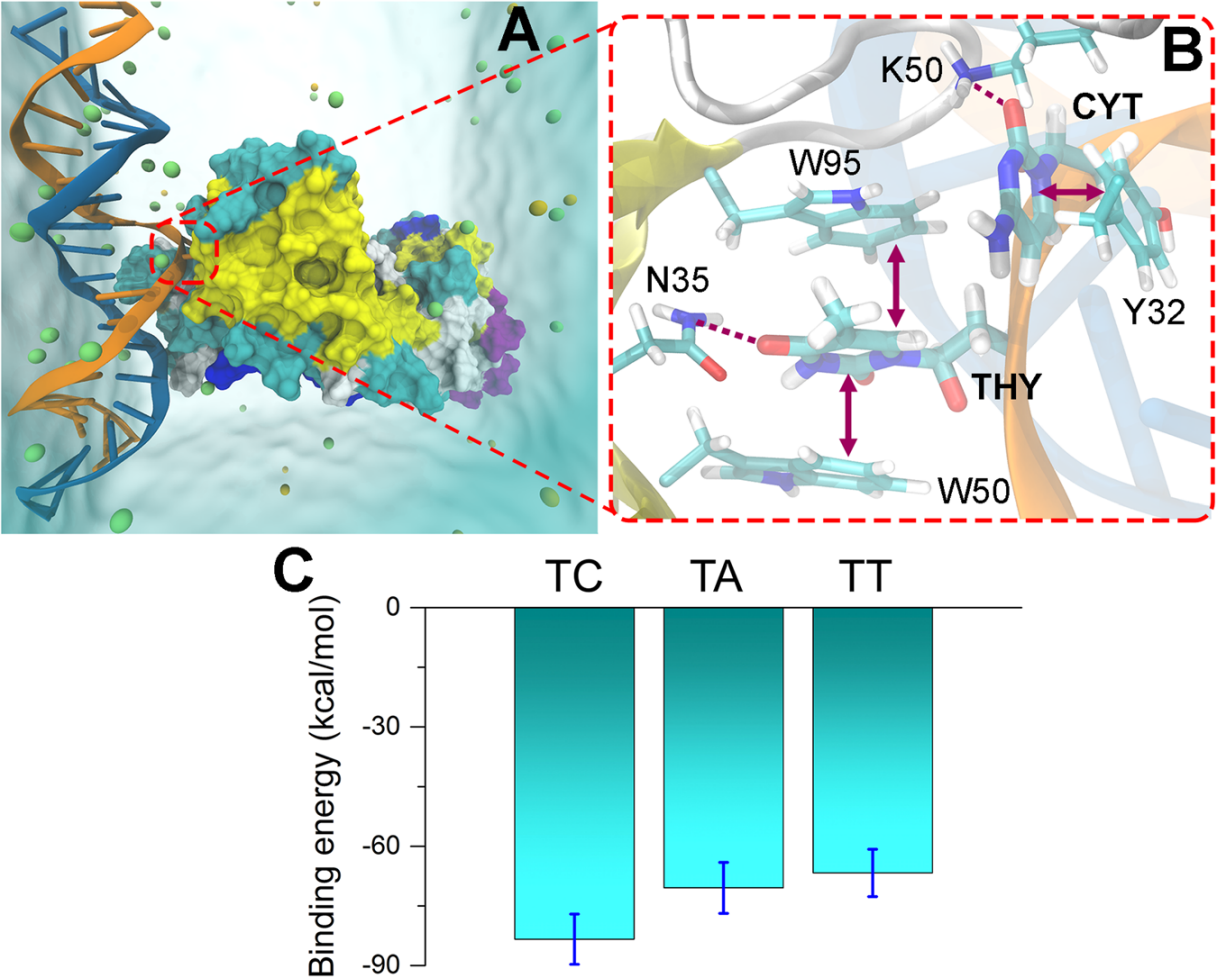
dsDNA binding to an antibody. A molecular representation of a dsDNA (blue and orange) bound to the ED-10 antibody, PDB ID 2OK0 (colored surface), derived by Molecular Dynamics simulations. The simulated system is solvated in a box of water with NaCl ions (spheres), as described in Methods. (**B**) A close-up view of the dsDNA-antibody binding site, featuring the base pair, dominantly interacting with several amino acid residues (labeled) through stacking interactions (arrows), and hydrogen bonds (dashed lines). (**C**) The interaction energy of the antibody with DNA was time averaged over the 100 ns simulation. The error bars indicate the standard deviation computed during the averaging procedure. Note, that the computed values represent the interaction energy of the two nucleic acid side chains with the entire antibody complex.

Lastly, we used computational methods to analyze IgG binding to the synthetic nucleic acid antigens to develop a model of a-DNA/DNA interaction. To date, only a few crystal structures of antibody/DNA complexes have been published. Among these, the structure of ED-10, a ssDNA-binding monoclonal antibody, complexed with the dinucleotide has been solved^13^. Taking into account previously reported cross-reactivity of a-ssDNA and a-dsDNA antibodies^30^, we reasoned that both types of complexes could share binding mechanisms. Therefore, we utilized the ED-10 structure to model anti-dsDNA-antibody complexes. Recently, it was reported that aromatic interactions mediate the 5′-base specificity of the ssDNA-binding antibody ED-10^31^. In our model, ED-10 binds to base pairs within double helix, leading to partially unwound dsDNA (Fig. 5A).

To study binding specificity of the antibody for DNA, we mutated the initial base pair in the antigen to alternative variants (in total, 40 variants were tested). Based on molecular modeling, the bound nucleotides adopted a conformation in which the nucleobase was twisted away from the sugar moiety. The relative binding affinities of the three dsDNA molecules to the antibody were then studied through 100 ns molecular dynamics (MD) simulations; details on the simulation tools and the simulation protocol are provided in Methods. Figure 5B shows a typical binding mode of dsDNA to the ED-10 antibody. The predominant binding arises from the stacking interactions between thymidine and the W50 and W95 amino acid residues, the cytidine and Y32 (arrow in the figure). The hydrogen bonds between thymidine and N95, and cytidine and K50 (dashed lines in Fig. 5B) also stabilize the dsDNA-antibody complex.

The average interaction energies of the antibody with the diverse base pairs are shown in Fig. 5C. These binding energies represent the time-averaged value over 100 ns and are representative measures of the strength of the dsDNA binding to the antibody. The time-dependence of energies in the three simulations are shown in Fig. S11, which illustrates that the energy fluctuates steadily around some average value.

Based on the results of ELISA and molecular modeling, we concluded that antigen D5 was the most reactive in binding DNA in pediatric and adult SLE, and that the structural basis for the recognition involved both the stacking interactions and hydrogen bonding between TC dinucleotide repeat of D5 and amino acids in antibodies.

## Discussion

Previously, we and others observed recognition of synthetic DNA by monoclonal a-dsDNA antibodies^9,13,22^. In our present work, we took a next step towards better understanding of autoantibodies to nucleic acids and towards an improved assay using novel synthetic DNA molecules. As we show, these molecules were efficient antigens for quantitation of a-dsDNA using standard ELISAs. Compared to currently applied DNA antigens, the tests of SLE samples showed high reproducibility and specificity when synthetic DNA were used. The new antigens were also stable upon storage as individual molecules and after immobilization on microtiter plates (data not shown).

The major advantages of applying synthetic antigens are high homogeneity, controlled purity and most importantly, known sequence^22^. These factors allowed us for the first time to study a-DNA profiles to a panel of ss and ds antigens in patients diagnosed with pSLE and adult-onset SLE. According to our studies, SLE patients had overall higher titer of antibodies toward sequence specific antigens, and only few patients had antibodies toward ATCG-mixed ds analogues without a distinguished pattern. This differs from results with ANA+ polyJIA subjects; fewer polyJIA patients had a-DNA antibodies, and in all cases, these antibodies preferentially recognized mixmer ds antigen. None of JIA subjects had a-ssDNAs. Dose-response curves and studies of 21mer antigens additionally confirmed that target binding by a-DNA was sensitive to the nucleotide sequence of applied antigens.

Based on our results, it is possible that antibody reactivity toward D5 is a distinctive feature of SLE, with the highest activity in pediatric disease. One possible explanation for this could be the overexpression of D5 in SLE. However, the biological role of D5 and other sequence-controlled antigens requires more investigation. A combination of the methods described herein and of modern genomic technologies could be an exciting next step towards better understanding of a-DNA and their role in SLE.

Multiple healthy subjects had elevated titers of a-ssDNA, but not of a-dsDNA. This could be caused by coiling of the ss antigen into 3D shapes that may interact non-specifically^31^. Previously it was suggested that elevated a-ssDNA titers is a distinctive feature of drug-induced SLE (DISLE)^32^. As no DISLE causing medication was used by the SLE subjects, we studied, our data excludes association between a-ssDNA positivity with use of particular drugs. Nevertheless, our study implies that clinical value of a-ssDNA is low in SLE.

Currently, there are conflicting reports on correlation between a-dsDNA and other ANA with clinical phenotypes of autoimmune diseases^9,29^. Most consistently reported associations are lupus nephritis, total disease activity index and flares in SLE, and chronic uveitis in oligoarticular JIA^9,33–35^. In this study, we hypothesized that sequence specific antibodies might correlate with a different subset of clinical phenotypes and help determine subgroups of patients based on their a-DNA status. We focused on several aspects of increased antibody titers: correlation with other biomarkers or treatment at a single time-point (disease onset), and correlation with flares during the treatment course. Generally, high titers of antibodies toward synthetic DNA correlated with high disease activity at onset as determined by SLEDAI^36^. However, we found no correlation with other biomarkers including ANA, complement or anti-Smith antibodies. a-DNA reactivity toward difference oligonucleotide sequences also varied for individual SLE subjects with active disease.

A recent report describes molecular subgroups in pSLE according to transcriptional analysis^37^. Notably, the strongest correlations in this study were between molecular signatures and anti-ds DNA and between signatures and disease activity by SLEDAI. We speculate that a larger panel of synthetic nucleic acids might allow higher resolution of molecular subsets in a clinically practical way.

In most pSLE patients, upcoming exacerbations of disease were not clearly predicted by changes in common serological tests, including ANA. Clinically tested a-dsDNA levels decreased along with the treatment, but they did not rise prior to flares. However, increased titers of IgG antibodies to antigens were detected in all a-DNA positive patients used in this study prior to flares.

Computational analysis of binding between short dsDNA antigens and monoclonal antibody shed light on the molecular basis of recognition. Binding free energies for novel dsDNA antigen and ED-10 were computed from classical all atom MD, employing the computational software NAMD^38^, and further analyzed in program VMD^39^. The obtained binding energies correlated with ELISA results of plasma samples using antigens D1-D3, and the most stable binding was observed for the TC rich dsDNA. In the initial X-ray structure adopted for the simulations, the ED-10 antibody selectively bound to DNA. According to our results, binding of ED-10 to the internal part of dsDNA stabilized complexes for a simulation period over 100 ns. This implies structural similarity for the original ssDNA‒ED-10 interaction and synthetic dsDNA-antibody interactions.

## Conclusion

Overall, synthetic antigens described herein demonstrate high specificity, sensitivity and reproducibility in detection of a-DNAs in SLE, a disease known to be associated with a-DNAs, and for the first time enable a detailed structural study of sequence specificity of these autoantibodies. Other important advantages of the new synthetic antigens compared to natural heterogeneous molecules are: (1) Known specificity, including easily controlled sequence-specific binding of a-DNA antibodies; (2) Potential to determine individual antibody profiles which may have clinical implications; and (3) Potential to determine the biological role of a-DNA in SLE. Thus, rationally designed nucleic acids might become a basis for development of standard clinical and scientific assays for SLE and other autoimmune conditions where ANAs have been detected, such as mixed connective tissue disease and scleroderma. Detection of sequence-specific a-DNA cannot be applied alone; however, these assays could become a valuable supplement to existing laboratory tests and analysis of clinical manifestations, with the aim of improving diagnostics and treatment of autoimmune diseases.

## Methods

### Patients

Human samples (60 healthy controls (SU+OUH), 40 pSLE sera from 27 subjects (SU), 213 adult SLE sera (OUH), 31 adult SLE sera (SU), and 14 ANA+polyJIA plasma) were provided by Stanford University School of Medicine (SU), USA, and Odense University Hospital (OUH), Denmark. The samples were characterized by clinical data, common serological biomarkers, treatment intensity and disease activity, see Supplementary Information.

Written approval by The Danish Data Protection Agency was obtained in November 2015. Committee of Information Safety of the Region Southern Denmark at the Danish Data Protection Agency specifically approved the whole study (permission signed by Birger Møller; Project ID: S-20080097). The methods were carried out in accordance with the relevant guidelines and regulations as stated in the Act on Processing of Personal Data adopted by the Danish Data Protection Agency on June 2^nd^ 2000. Personal data of patients was not used in this work. Therefore the informed consent from the individuals was not needed.

### Reagents

CTD was purchased from Sigma. The sequences were designed using in house design software in order to avoid hairpin formation and self-annealing by individual strands.

DNA strands were designed and synthesized at the University of Southern Denmark by solid-phase phosphoramidite method. Their structure and purity was confirmed by MALDI MS and IC HPLC, respectively.

### DNA antigens: D1

(5′-TGCACTCTATGTCTGTATCAT-3′): (5′-ATGATACAGACATAGAGTGCA-3′); **D2**, (5′-TCCTCTCTTTCTCTTTCTCTT-3′): (5′-AAGAGAAAGAGAAAGAGAGGA-3′); **D3**, (5′-ATTTATTTTTATATTTATATT-3′): (5′-AATATAAATATAAAAATAAAT-3′); **D4**, (5′-CATGAAGACCTCACAGTAAAAATAGGTGATTTTGGTCTAGCTACAGTGAAATCTCGATGGAGT-3′): (5′-ACTCCATCGAGATTTCACTGTAGCTAGAC CAAAATCACCTATTTTTACTGTGAGGTCTTCATG); **D5**, (5′-TCCTCTCTTTCTCTTTCTCTTTCCTCTCTTTCTCTTTCTCTTTCCTCTCTTTCTCTTTCTCTT-3′): (5′-AAGAGAAAGAGAAAGAGAGGAAAGAGAAAGAGAAAGAGAGGAAAAGAAAGAGAAAGAGAGGA-3′); **SD1**, 5′-CATGAAGACCTCACAGTAAAAATAGGTGATTTTGGTCTAGCTACAGTGAAATCTCGATGGAGT-3′; **SD2**: 5′-TCCTCTCTTTCTCTTTCTCTTTCCTCTCTTTCTCTTTCTCTTTCCTCTCTTTCTCTTTCTCTT-3′.

For annealing, DNA strands were mixed at a molar ratio 1:1 in 1X PBS buffer (Life Technologies, pH 7.2), kept at 90 °C for 10 min followed by cooling to room temperature over 4 h.

### ELISA

Maxisorb 96 well plates (NUNC Thermofisher) were coated with ss/ds antigens at concentration 2 µg/mL in 1X PBS overnight (RT; 150 µl/well). After washing with 1X PT (2 × 300 µl/well, PT: 50 µl Tween-20 in 1 L 1X PBS), the plates were blocked with 1X PTB (1 h, 37 °C; 100 µl/well, PTB: 20 g BSA, 50 µl Tween-20 in 1 L 1X PBS). Incubation with plasma at desired dilution was performed at 37 °C for 1.5 h using diluent: 2 g BSA, 50 µl Tween-20 in 1 L 1X PBS (100 µl/well). This was followed by washing (2 × 300 µl 1X PBS) and incubation with HPR-labelled secondary antibody for 1.5 h at 37 °C using same diluent and dilution of the secondary antibody provided by supplier (HPR-conjugated a-aIgG or a-IgM; Sigma). Subsequent washing (2 × 300 µl PT) and incubation with freshly prepared TMB-H_2_O_2_ solution (Sigma; 100 µl/well) was followed by adding a stop solution (1 M H_2_SO_4_; 50 µl/well) and reading resulting absorbance values at 450 nm on Magellan Tecan microplate reader. Linear range for each antigen was determined via testing series of control dilutions (HNP, HSS, HDD in dilutions 1:50 to 1:2000). According to the results plasma dilutions 1:100 - 1:500 were within linear range of the assay for each antigen (R^2^ > 0.95).

### Bradford assay

Total amount of protein in plasma samples was estimated by Bradford method using standard curve of BSA control at known concentration^40^. In a maxisorb 96 well plate controls (BSA standard samples at concentrations 2 mg/mL, 1 mg/mL, 0.5 mg/mL and 0.1 mg/mL) and plasma sample were mixed with a Bradford reagent following manufacturer’s protocol (Biomed). Plasma samples were used in dilution 1:100. Resulting absorbances at 595 nm were measured on Magellan Tecan microplate reader. Total amount of protein was calculated using standard curve.

### Antibody titration curves

Prior to analyses, each sample was characterized by A_450_ value using IgG ELISA assay. The assay was carried out using antigens D4, D5, CTD, SD2, and 1:100 plasma/sera dilution under the conditions described above. Afterwards, sample dilution values were re-calculated for each antigen in order to obtain similar absorbance per total sample protein. The obtained dilution was used as a highest concentration for further serial dilution. The obtained samples were analyzed using similar IgG ELISA setting as described above.

### Statistical analyses

Are described in detail in the Supplementary Information. Differences were analyzed for statistical significance with OLS and ANOVA in R^26,28,41^. A P value of less than 0.05 was considered significant.

### Molecular dynamics simulations details

The binding of three modifications of the DNA 21-mer to the monoclonal antibody ED-10 (PDB ID: 2OK0)^13^ were studied using the classical molecular dynamics (MD) approach. MD simulations were performed using NAMD 2.9^38,42^ with the CHARMM22 force field for nucleic acids and proteins with CMAP corrections^42,43^ and the TIP3P water model. In all simulations the antibody-DNA complex was neutralized by a 50 mM solution of NaCl. Periodic boundary conditions were adopted in all MD simulations and the particle-mesh Ewald (PME) summation method was employed for evaluating Coulomb forces. The van der Waals (vdW) energy was calculated using a smooth cutoff of 12 A. The integration time step was 2 fs; temperature was kept at 310 K by applying Langevin forces with a damping coefficient of 5.0 ps^-1^ to all atoms in the system, except hydrogens.

Each simulated system was first energy-minimized, then heated to 310 K. The simulation protocol was similar to the one employed in earlier studies^44–46^, however, slightly different in the three cases. After heating, simulated system (i) was first equilibrated for 10 ns with harmonic restraints applied to the protein, and the dinucleotide basepair fixed in space as in the crystal structure. Next, the basepair was released, while the protein was still harmonically restrained, and the system was simulated for further 10 ns. Finally, all atoms were allowed to move and further 12.5 ns of simulations were performed under NPT ensemble conditions and using Nos´e-Andersen Langevin piston pressure control^44–46^, allowing the systems to acquire a constant volume at 1 atm pressure. After equilibration, a 100 ns MD simulation was carried out in the NVT ensemble that was used for analysis. Note that the root mean square displacement (RMSD) calculated for all atoms of the antibody proteins, see Fig. S1, showed that the performed equilibration was sufficient to ensure a stable antibody structure.

In the case of systems (ii) and (iii), the pre-equilibrated structure of system was used for the basepair mutations. The obtained mutants were then further equilibrated for 3 ns each, before a 100 ns production run for each system was carried out. The molecular mutations and structure analysis was performed with VMD^47^.

### Data availability

The authors declare that all other data supporting the findings of this study are available within the paper and its supplementary information files.

## Electronic supplementary material

Supplementary information
