# Supplementary material for: Autoantibody Profiling in Lupus Patients using Synthetic Nucleic Acids
Source: Sci Rep. 2018 Apr 3;8:5554. doi: 10.1038/s41598-018-23910-5 (PMC5883037; doi:10.1038/s41598-018-23910-5)
Supplement: Supplementary file 1 — Supplementary information [file 41598_2018_23910_MOESM1_ESM.pdf]

Autoantibody Profiling in Lupus Patients using Synthetic Nucleic Acids

(Supplementary Information)

Martin Klecka[1], Christina Thybo[2], Claudia Macaubas[3], Ilia Solov'yov[2], Julia Shimard[4], Imelda Maria Balboni[5], Emily Fox[5], Anne Voss[6], Elizabeth D. Mellins[3*] and Kira Astakhova[1*]

[1] Department of Chemistry, Technical University of Denmark, Kemitorvet 206, 2800 Kgs. Lyngby, Denmark

[2] Department of Physics, Chemistry and Pharmacy, University of Southern Denmark, Campusvej 55, 5230 Odense M, Denmark

[3] Department of Pediatrics, Program in Immunology, Stanford University School of Medicine, 269 Campus Drive, Stanford, California, 94305 USA

[4] Department of Health and Research Policy, Stanford University School of Medicine, 150 Governor's Lane, Stanford, California, 94305 USA

[5] Department of Pediatrics, Division of Allergy, Immunology, and Rheumatology, Stanford University, 700 Welch Rd. Suite 301, Stanford, California, 94304 USA

[6] Department of Rheumatology, Odense University Hospital, J. B. Winsløws Vej 19, 2. 5000 Odense C, Denmark

Table of contents

Contents

List of abbreviations 2

S1. ELISA assay establishment 4

S2. Demographic and clinical characteristics of subjects used in this study 8

S3. Statistical analyses 13

S4. Results of ELISA assay 18

S5. Data used in longitudinal study of pSLE subjects 22

S6. Detailed protocols for molecular dynamics 24

List of abbreviations

General

pSLE – pediatric systemic lupus erythematosus

polyJIA – polyarticular juvenile idiopathic arthritis

SLEDAI – systemic lupus erythematosus disease activity index

IgG – immunoglobulin class G

IgM – immunoglobulin class M

ELISA – enzyme-linked immunosorbent assay

HPR – horseradish peroxidase

CTD – calf thymus DNA

nt – nucleotide

MD – molecular dynamics

Biomarkers:

APL – antiphospholipid antibodies

WBC – white blood cell count

ANA – antinuclear antibody

RF – rheumatoid factor

a-DNA (ss/ds) – antibody against DNA (single-stranded/double-stranded)

Treatment

NSAIDS – nonsteroidal anti-inflammatory drugs

HCQ – hydroxychloroquine

IS – immunosuppressive therapy (cyclophosphamide, mycophenolate, or methotrexate)

MTX – methotrexate

PO steroids – oral steroids

IV steroids – intravenous steroids

Pred – prednisone

S1. ELISA assay establishment

To establish the ELISA assay, we used commercially available (Immunovision) human plasma containing high titers of polyclonal a-ssDNA (HSS) or a-dsDNA (HDD and a human monoclonal antibody recognizing CTD (Abcam)) as positive controls for autoantibody binding to the antigens. As a negative control, human normal plasma from a healthy individual (HNP) also was used. Plasma titration experiments verified binding to the panel of antigens by dilutions (1:100 - 1:500) of the polyclonal controls, HSS and HDD; see Supplementary Fig. S1 (Suppl. Section S1). In the assay measuring bound IgG, absorbance at 450 nm decreased linearly in the plasma dilution range 1:100 - 1:500, indicating specific binding to all antigens. Specificity was also confirmed by the absence of signal for HNP. In addition, no significant signal was obtained for any antigen when diluent alone was applied to plates pre-coated with antigen (data not shown). Less antigen and shorter incubation times were required for synthetic antigens, compared to CTD, with slightly higher signal for longer DNA analogues (> 60 nt). In addition, we saw no variation between levels of aDNA binding with different batches of antigen (not shown).

Stability of the synthetic antigens and reproducibility of the antibody reactivity are important metrics for diagnostic tests. This was studied by series of independent ELISA experiments using randomly selected pSLE samples (n = 15) over an 8 month period when the samples were stored at -78 °C. During this period, the pre-coated microtiter plates were stored at +4 °C and analyzed every 2 weeks. Results were highly reproducible for all the synthetic antigens, with deviation in A_{450} of 1-2%, whereas CTD showed decreased signal upon storage (12% after second month and 23% after third month; data not shown).

For the cutoff establishment, the following results were used (antigen/mean A_{450} value for healthy controls, n= 16):

D4/0.24, D5/0.27, CTD/0.29, SD1/0.53, SD2/0.68, D1/0.19, D2/0.30, D3/0.22.

Supplementary Figure S1: Plasma titration curves for polyclonal controls (IgG and IgM).

A) IgG

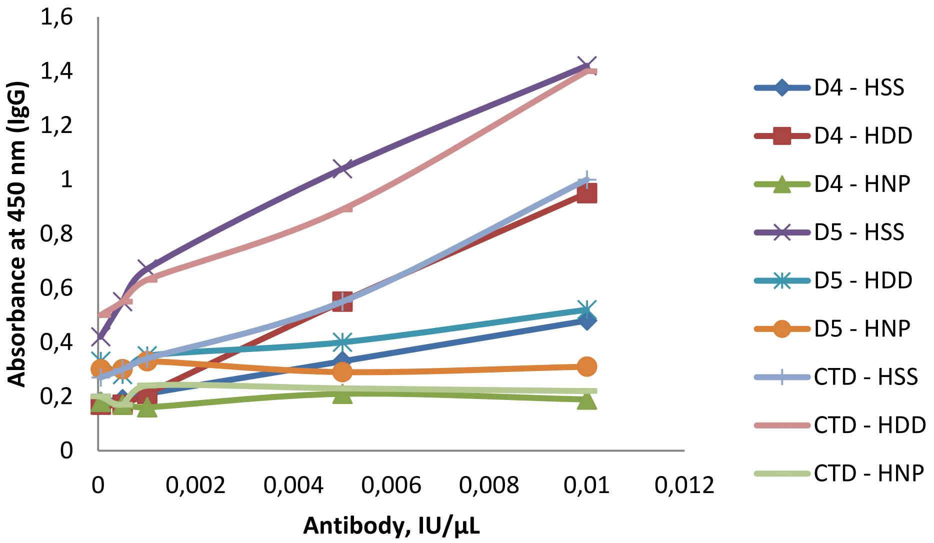
1,6
1,4
1,2
1
0,8
0,6
0,4
0,2
0
Absorbance at 450 nm (IgG)
0
0,002
0,004
0,006
0,008
0,01
0,012
Antibody, IU/µL
D4 - HSS
D4 - HDD
D4 - HNP
D5 - HSS
D5 - HDD
D5 - HNP
CTD - HSS
CTD - HDD
CTD - HNP

B) IgM

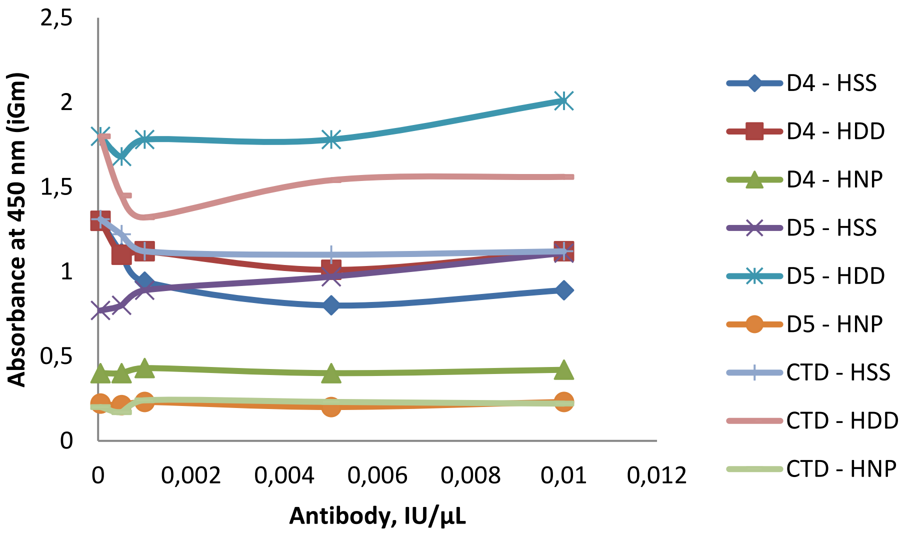
2,5
2
1,5
1
0,5
0
Absorbance at 450 nm (iGm)
0
0,002
0,004
0,006
0,008
0,01
0,012
Antibody, IU/µL
D4 - HSS
D4 - HDD
D4 - HNP
D5 - HSS
D5 - HDD
D5 - HNP
CTD - HSS
CTD - HDD
CTD - HNP

Supplementary Figure S2: Evaluation of binding of polyclonal controls and patient samples to the non-coated plate. Data are shown for randomly selected group of patients.

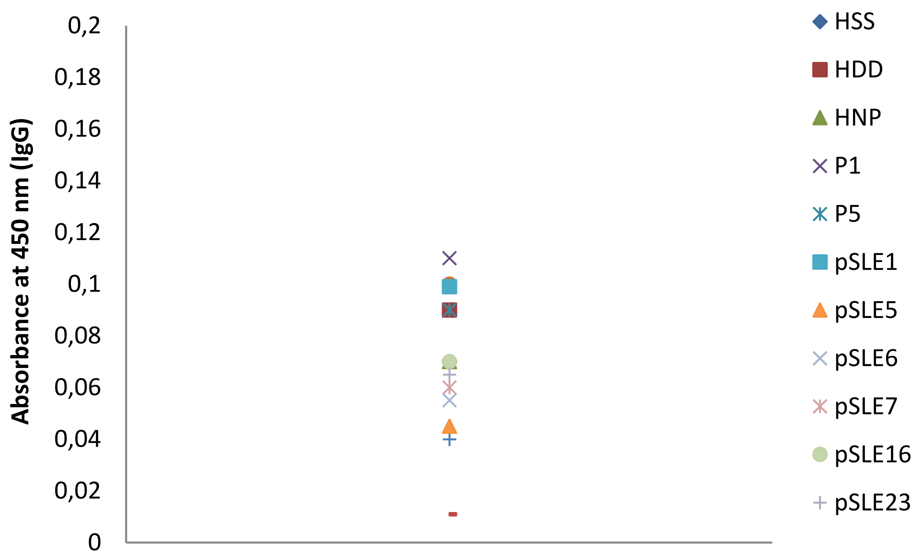

Supplementary Figure S3: Binding equilibrium time study for polyclonal controls and randomly selected patient samples with antigens used in this study.

A)

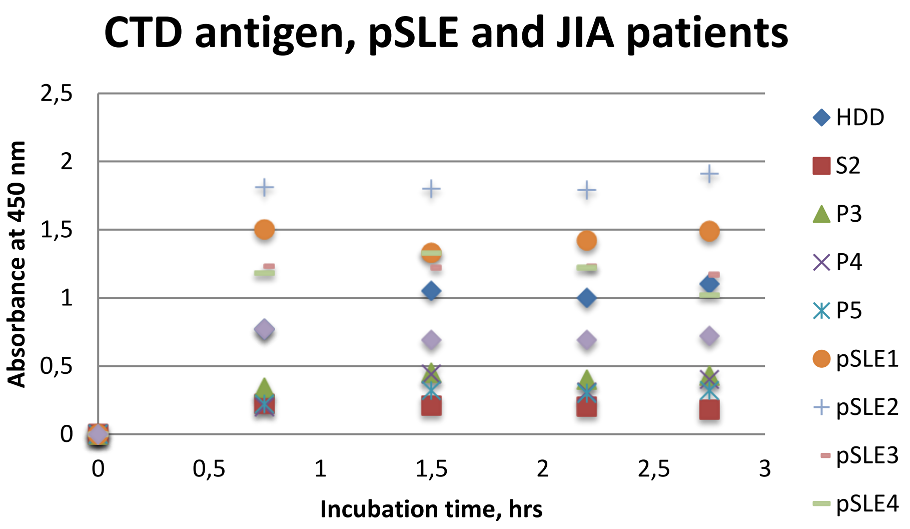

B)

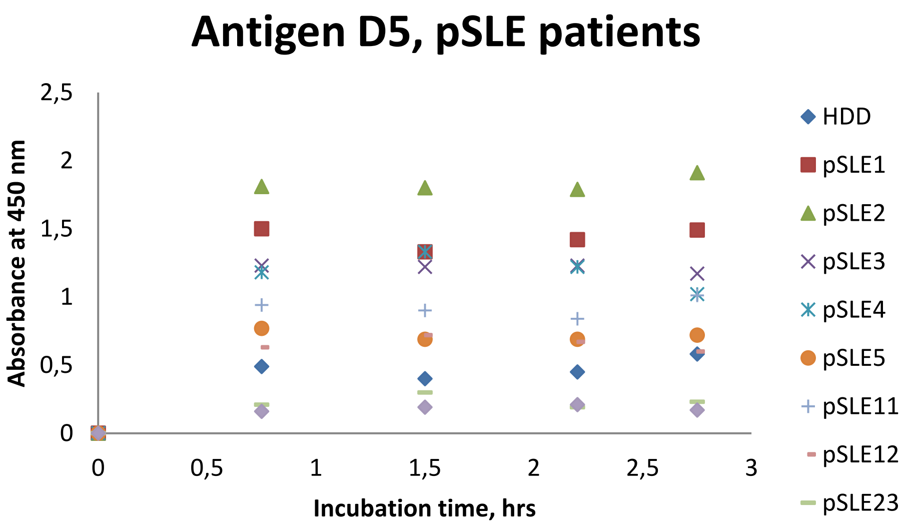

S2. Demographic and clinical characteristics of subjects used in this study

At sample collection, median age of the pSLE patients was 14 years and median disease duration was 4 months. For adults, median age at sample collection was 38 (SU) and 46 (OUH), with 18 years from the disease onset (median value). By clinical laboratory testing, all SLE and polyJIA subjects were ANA positive. Furthermore, 12 (44%) pSLE, 39% adult SLE (SU) and 42% adult SLE (OUH) were a-DNA positive by *Crithidia* substrate and ELISA (Table S1). Other clinical parameters collected on pSLE patients included ESR, complement C3 and C4, titers of anti-Smith antibodies, antiphospholipid antibodies and kidney biopsy in appropriate patients.

Prior to testing patient samples in the ELISA, we measured total protein by Bradford assay (26), and samples were adjusted to equal total protein concentrations (50 µg/mL).

Supplementary Table S1: Demographic and clinical characteristics of SLE subjects (pediatric and adult SLE, Stanford University Hospital; adult SLE, Odense University Hospital).

**Variable**	**pSLE subjects (n=27), Stanford**	**Adult SLE (n=31), Stanford**	**Adult SLE (n=212), Odense**
Female/male	24/3	27/4	191/21
Hispanic (%)	16 (59)	6 (19)	0 (0)
Asian/Pacific Islander (%)	5 (19)	6 (19)	0 (0)
Non-Hispanic Caucasian (%)	6 (22)	6 (19)	212 (100)
African-American (%)	0 (0)	4 (13)	0 (0)
Age at diagnosis, median (range) years	14.1 (7.5-17.6)	24 (2-44)	34 (10-78)
Age at sample collection, median, (range) years	14.5 (7.5-19.2)	38 (20-68)	46 (20-84)
dsDNA antibody positive patients at onset – *Crithidia assay (*) or ELISA (**)* (%)	12 (44)*	12 (39)*	89 (42)**
Class III/IV lupus nephritis-biopsy proven† (%)	10 (37)	nd	12 (5.6)
Mean (range) SLEDAI	SLEDAI 11.6 (4-30)	SLEDAI 3.2 (0-8)	3.4 (0-28)
Mean (range) ESR	48 (1-132)	nd	16 (2-51)
Mean (range) C3 complement	72 (24-121)	91.4 (26-141)	1 (0.23-6)

Treatment at sample*:			
PO steroids (%)	13 (48)	20 (64.5)	nd
IV steroids (%)	7 (26)	0 (0)	nd
HCQ (%)	17 (63)	22 (71)	nd
Other IS (%)	4 (15)	18 (58)	nd
Daily NSAID (%)	6 (22)	6 (19)	nd
None	2 (7)	0 (0)	nd

For symbols, see Abbreviations. nd = no data.

Supplementary Table S2: Demographic and clinical characteristics ANA+ polyJIA and healthy control subjects.

**Variable**	**ANA+ PolyJIA**	**Healthy control (HC)**
N (subjects)	14	60
Female/Male	12/2	44/16
African-American	0	0
Asian/Pacific islander	0	5
Caucasian	2	51
Caucasian Hispanic	8	2
Ethnicity unknown	4	2
Median age (yr) at disease onset (range)	7 (1-15)	na
Median age (yr) at sample collection (range)	12 (7-20)	27 (12-39)
Fever/total no. samples (%)	na	na
Rash/total no. samples (%)	na	na
Median joint count (range)	8 (0-46)	na
Median WBC ($x10^3$/ul) (range)	8.2 (5.9-12.3)	na
Median platelets ($x10^3$/ul) (range)	314 (165-666)	na
Median ESR (mm/h) (range)	5 (1-28)	na
Median prednisone dose, mg/kg/day (range)	0 (0-0.05)	na
NSAIDS/total no. samples (%)	8 (57%)	na
Methotrexate/total no. samples (%)	4 (28%)	na
Anti-TNF /total no. samples (%)	12 (85)	na
IL-1RA /total no. samples (%)	0 (0%)	na
Other medications: HCQ	1 (7%)	na

na = not applicable

Supplementary Table S3: Clinical parameters of pSLE patients grouped according to a-D5 and a-CTD IgG titers.

**Variable**	**a-D5+ positive**	**a-D5+ elevated**	**a-D5- negative**	**a-CTD+ positive**	**a-CTD+ elevated**	**a-CTD- negative**
N (subjects)	12	4	11	9	9	9
Female/Male	10/2	4/0	9/2	7/2	9/0	8/1
Median age (yr) at disease onset (range)	14 (10-17)	11 (7-14)	14 (9-17)	14 (11-17)	12 (9-15)	13 (7-17)
Median age (yr) at sample collection (range)	14 (10-17)	11 (7-14)	14 (9-17)	14 (11-17)	13 (9-19)	14 (7-17)
dsDNA antibody positive patients (%)	6 (50)	1 (25)	1 (9)	9 (100)	9 (100)	0 (0)
ANA+ (%)	12 (100)	4 (100)	11 (100)	9 (100)	9 (100)	9 (100)
RNP + (%)	6 (50)	2 (50)	1 (9)	5 (55)	1 (11)	3 (33)
Ro + (%)	2 (17)	2 (50)	1 (9)	4 (44)	1 (11)	1 (11)
Smith + (%)	6 (50)	2 (50)	2 (18)	5 (55)	1 (11)	4 (44)
Class III/IV lupus nephritis-biopsy proven† (%)	7 (58)	2 (50)	1 (9)	7 (77)	4 (44)	2 (22)
Arthritis + (%)	8 (67)	4 (100)	5 (45)	7 (77)	4 (44)	5 (55)
Renal + (%)	6 (50)	2 (50)	3 (27)	6 (66)	4 (44)	1 (11)
Mean (range) SLEDAI score	15.5 (6-30)	12.5 (4-30)	5 (2-8)	20.5 (10-30)	7 (2-17)	6 (0-14)
APL positive (%)	42	1 (25)	3 (27)	2 (22)	3 (33)	2 (22)
Mean (range) ESR	70 (6-132)	44 (0-119)	29 (6-110)	85 (60-132)	18 (7-50)	36 (9-110)
Mean (range) C3	57 (18-120)	68 (28-95)	93 (71-115)	38 (18-87)	85 (33-121)	96 (71-132)
Treatment at sample*:						
PO steroids (%)	7 (58)	4 (100)	4 (36)	3 (33)	7 (77)	5 (55)
IV steroids (%)	3 (25)	0 (0)	2 (18)	4 (44)	1 (11)	1 (11)
HCQ (%)	8 (67)	3 (75)	5 (45)	4 (44)	7 (77)	6 (66)
Other IS (%)	4 (33)	2 (25)	4 (36)	2 (22)	1 (11)	2 (22)

Supplementary Table S4: Results for a-DNA IgG ELISA assay using new antigens and clinical data for randomly selected individual pSLE patients.*

Patient Dis, #	a-D5	a-D4	a-CTD	Age S/O	Gender	SLE-DAI	ANA	C3	APL	aDNA (C)	Sm	*A*	*R*
**a-D5++ (strongly positive)**													
pSLE1	++	+	++	14/13	F	8	+	98	+	+/-	-	Y	N
pSLE3	+++	-	+/-	11/11	F	4	+	86	+	+	-	Y	N
pSLE5	++	++	+/-	16/16	F	7	+	71	+	-	+	Y	N
pSLE7	++	++	-	15/15	M	27	+	85	-	+++	+	Y	Y
pSLE8	++	+	++	13/13	M	25	+	18	-	+++	+	N	N
pSLE9	+++	++	+++	16/16	F	30	+	25	-	+++	+	Y	Y
pSLE10	++	++	+	12/12	F	26	+	24	-	+++	+	Y	Y
**a-D5+ (positive)**													
pSLE11	+	-	+	12/12	F	6	+	62	+	+	-	Y	N
pSLE12	+	+	++	14/14	F	30	+	28	-	++	-	Y	N
**a-D5- (negative)**													
pSLE15	-	-	-	16/11	F	6	+	91	-	-	-	Y	N
pSLE20	-	-	-	15/15	M	6	+	104	-	-	nd	N	N
pSLE21	-	-	+/-	10/10	F	2	+	81	nd	+	nd	nd	nd
pSLE22	-	-	-	15/15	F	0	+	120	-	-	-	Y	N
pSLE23	-		+	17/16	F	4	+	83	nd	+	nd	Y	Y
pSLE24	-	-	-	16/16	F	0	+	132	+	-	-	Y	N

* **Patients:** 15 subjects were selected randomly out of entire cohort (n = 27) using MatLab; Mean value of a-D4 titer for the random group was similar to the mean value for the entire cohort (A_{450} 0.98 and 0.99 per total sample protein 55 mg/mL, respectively).

Abbreviations: Dis = disease, Age (S/O) = full years at sample and disease onset; ANA = antinuclear antibodies, C3 = complement C3, APL = anti-phospholipid antibodies; aDNA (C) = antibodies toward DNA detected in clinical laboratory, Sm = Smith, A = arthritis, R = renal; nd = not determined.

Cut-off values: Signal: +++, ++, + and +/- corresponds to 5x, 4x, 3x and 2x standard deviation above mean value of A_{450} for healthy controls using each antigen.

S3. Statistical analyses

To test the normality QQ plots were constructed (Figure below and Figure S4 in the Supporting Information). Afterwards, Shapiro-Wilk W test for normal data confirmed p>z over 0.05 (ref. 3). In short, to reject the 0-hypothesis (the variable is normally distributed) at a significance level of a, when the probability p>z values are smaller than a. Thus, according to the Shapiro-Wilk test, at a 5% significance level, we cannot reject the normality of the groups.

Supplementary Figure S4. QQ plots for the observations.

D4 pSLE

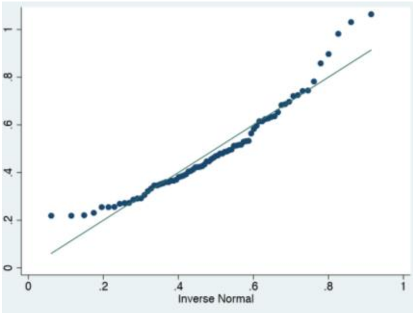

D4 SLE adults

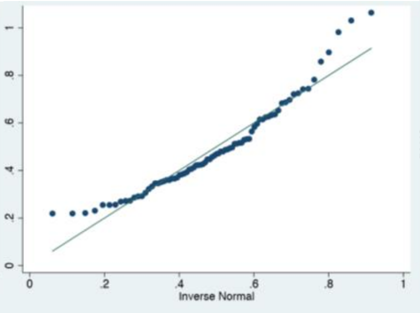

D4 HC

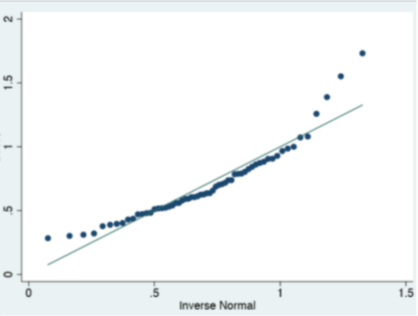

D5 pSLE

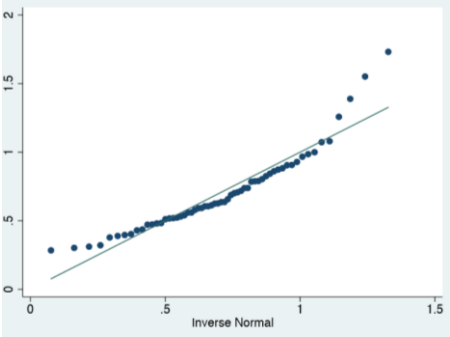

D5 SLE adults

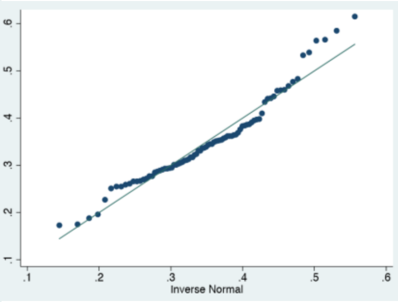

D5 HC

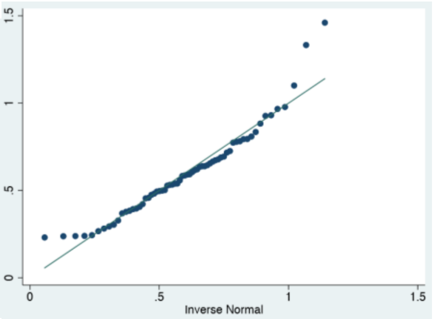

Ctd pSLE **Ctd SLE adults** **Ctd HC**

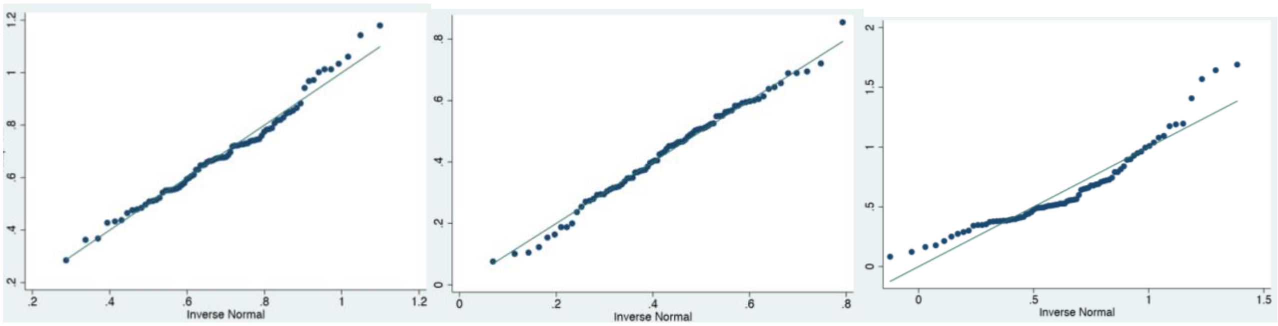

Statistical analyses against multiple variables were carried out using OLS in R. The rationale for grouping the patients was the observed positive or negative interaction with corresponding antigens in IgG ELISA experiments. Thus, to run the test, patient cohorts were separated into the following groups:

pSLE and SLE, D4 elevated and negative

pSLE and SLE, D5 positive, elevated and negative

pSLE and SLE, CTD positive, elevated and negative

HC, D4 and D5 negative

Next, we indicated binary parameters for statistical analyses from the clinical data of the patients:

gender (F/M), ethnicity Asian/ Pacific Islander (+/-), ethnicity Hispanic (+/-), ethnicity Non-Hispanic Caucasian (+/-), SLEDAI (>10/<10), clinical a-dsDNA test (+/-), APL (+/-), C3 (>50/<50), Smith (+/-), arthritis (+/-), renal (+/-).

We also selected the following medications (prior and at sample), as binary parameters for the t-test:

PO steroids (+/-), IV steroids (+/-), HCQ (+/-), other IS (+/-), NSAID (+/-), none (+/-).

OLS tests for the groups were carried out in R. Using the Shapiro-Wilk test, we confirmed that the error in OLS test was normally distributed with $p>0.05$. This additionally confirms that the results of OLS are adequate (3).

Supplementary Table S5. Results of regression analysis by OLS in R – pSLE, Stanford University.

a-D4	a-D5	a-DNA clin	C3	anti-Smith+	a-RNP+	APL+	SLEDAI
0.7	1.11	0.5	98	0	0	1	8
0.45	0.89	0.7	86	0	0	1	4
0.78	1.34	0	71	1	0	1	14
0.82	0.99	1	85	1	0	1	27
0.66	1.32	1	18	1	1	1	25
0.55	2.11	1	25	1	1	0	30
0.78	1.50	1	24	1	0	1	26
0.78	1.80	0.7	28	0	1	1	30
0.21	0.39	0	91	0	0	0	6
0.34	0.40	0	104	0	0	0	6
0.43	0.54	0.7	62	0	1	0	6
**OLS result: a-D4**							
p value-Inercept		0.002019	0.000298	8.96E-05	5.99E-05	0.000274	0.00243
p value- X variable		0.131404	0.193608	0.05842	0.875265	0.003444	0.021597
**OLS result: a-D5**							
p value-Inercept		0.030811	3.52E-05	0.002066	0.001054	0.011772	0.048979
p value- X variable		0.076673	0.006403	0.075051	0.167198	0.252635	0.00209

Supplementary Table S6. Linear regression results (P values X variable) for adult SLE, Stanford University (SU) and Odense University Hospital (OUH).

	SU/OUH result		
Y/X input	a-D4	a-D5	a-DNA clin
a-D4	-	0.52/0.42	0.67/0.52
a-D5	0.52/0.42	-	0.51/0.21
a-DNA clin	0.67/0.52	0.51/0.21	-
C3	0.71/0.70	0.68/0.74	0.009/0.41
Anti-Sm+	0.77/0.65	0.49/0.40	0.39/0.63
a-RNP+	0.13/0.20	0.10/0.29	0.23/0.17
SLEDAI	0.52/0.49	$0.0008/1.6*10^{-11}$	0.78/0.015

Group comparison [3,4]

To analyze the difference between mean antibody titers for patient groups, we applied one-way ANOVA in Stata, followed by multiple comparison test. Difference between the groups was statistically significant with p = 0.017.

Next, using three-way ANOVA test, we examined if there was an interaction between a-D4, a-D5 and a-CTD levels (three independent binary variables) and the disease activity across these groups (continuous dependent variable). pSLE had a significant one-way interaction with a-D5, p = 0.022, but no two- or three-way interactions (p>0.05).

Supplementary Table S7: Treatment details for randomly selected pSLE patients (n=15).*

**a-D5+:** Pat Dis #	Medication		**a-D5-negative:** Pat Dis,#	Medication	
	At sample	Previous treatment		At sample	Previous treatment
pSLE1	Pred, HCQ, Vioxx, ranitidine	IV Solumedrol (steroids)	pSLE15	Piroxicam	Piroxicam
pSLE3	Ibuprofen	none	pSLE20	Pred, HCQ, CellCept	Solumedrol, then Pred, Cytoxan, Solumedrol, CellCept
pSLE5	IV Solumedrol then Pred, HCQ	none	pSLE21	Pred, HCQ, ranitidine, IV Solumedrol and Cytoxan this visit	Pred, HCQ, then IV Solumedrol and Pred tapered
pSLE7	IV Solumedrol	none	pSLE22	Pred, HCQ, indomethacin, baby aspirine, ranitidine	Pred
pSLE8	IV Solumedrol, then Pred, HCQ	none	pSLE23	Pred, HCQ, baby aspirin	Pred
pSLE9	IV Solumedrol, Cytoxan	steroids	pSLE24	Plaquenil, baby aspirin, Topamax	Pred
pSLE10	IV Solumedrol	IV Solumedrol then Pred, IV Cytoxan			
pSLE11	Pred, HCQ, baby aspirin, ranitidine	naproxen, HCQ, Pred			
pSLE12	none	none			

The sample of 15 patients was selected randomly out of the entire group. Median a-D4 titer for the group was similar to the median value of the entire cohort (0.67 and 0.68).

S4. Results of ELISA assay

Supplementary Figure S5: Binding assay for determination of individual autoantibody profiles in patients. Bound autoantibodies recognize corresponding antigen in ELISA assay. Binding reactions were carried out at 37˚C with dilution series of patient serumin PBS. Two- to tenfold enhancement of absorbance was observed.

A) aD4 in pSLE samples

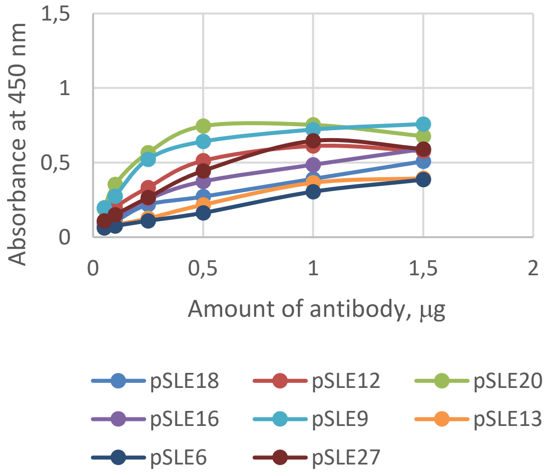

B) aD5 in pSLE samples

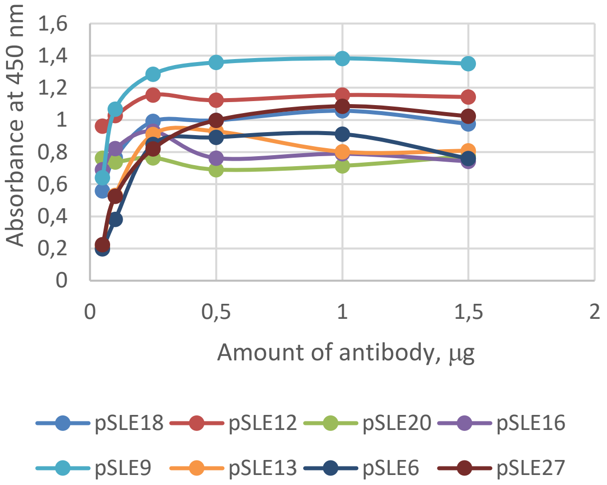

C) aCTD in pSLE samples

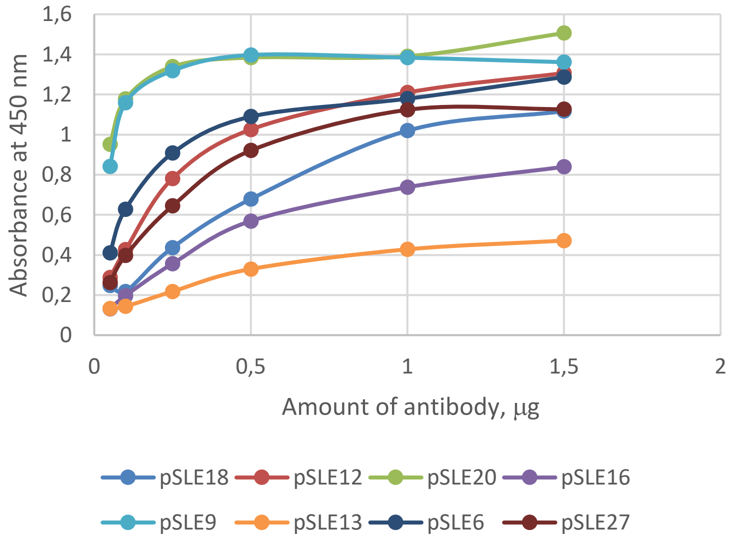

D) D4 – JIA samples

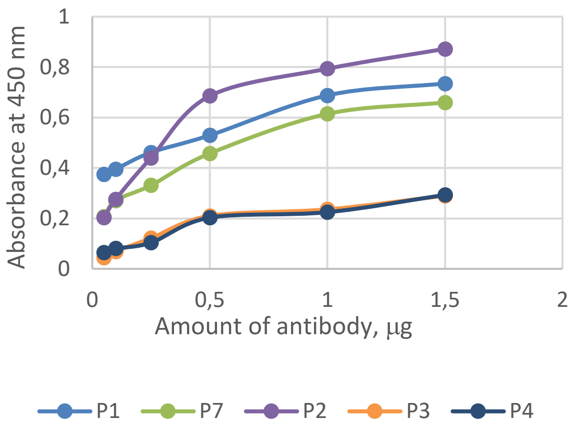

E) D5 – JIA samples

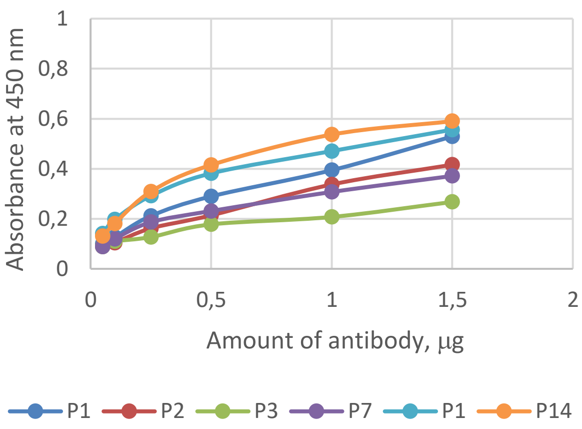

Supplementary Figure S6: Results of IgG ELISA assay using antigens D1-D3.*

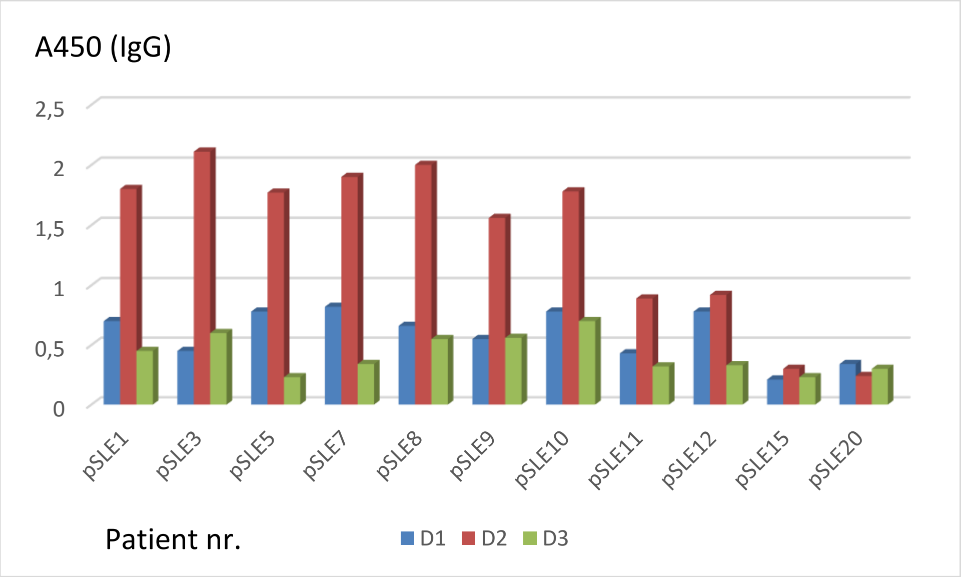

* ELISA assays were carried out using as described in Materials and Methods. Sequences of antigens D1-D3 are shown in Figure 1.

Supplementary Figure S7: Results of IgG ELISA assay for adult SLE using D4 and D5.*

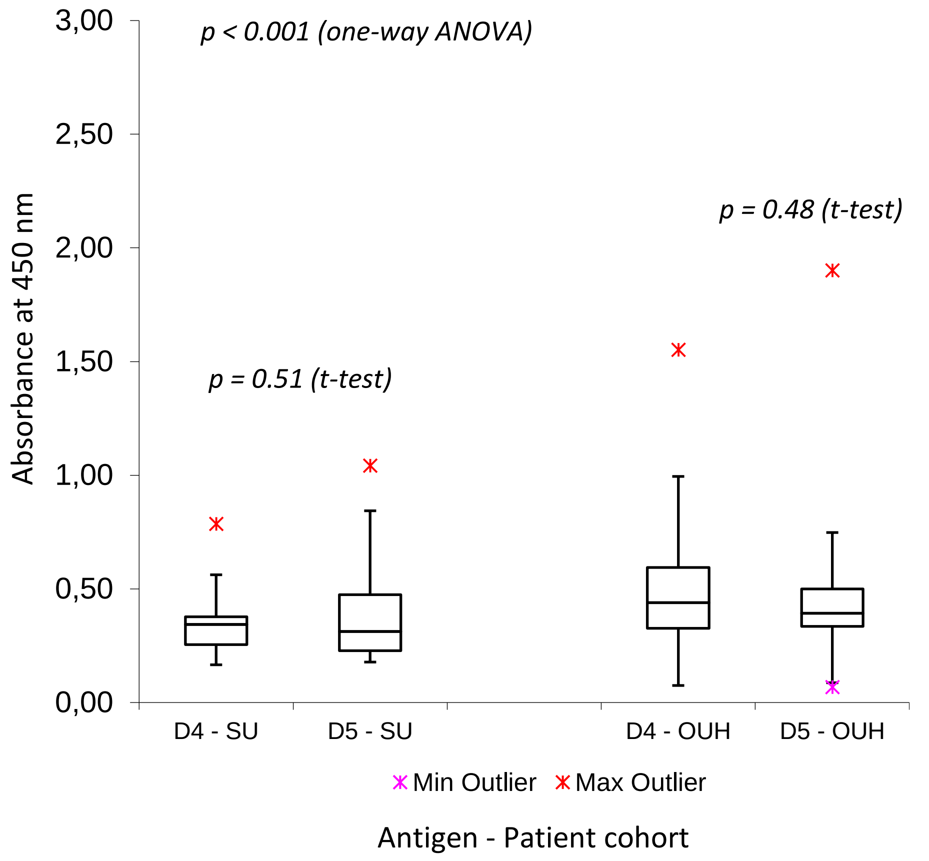

* ELISA assays were carried out using as described in Materials and Methods. Sequences of antigens D4-D5 are shown in Figure 1.

S5. Data used in longitudinal study of pSLE subjects

Supplementary Table S8: Parameters of pSLE patients in longitudinal study.

Patient/visit nr	Time after onset (months)	a-D5	a-CTD	SLEDAI	ANA	Sm	APL	a-RNP	C3	C4
pSLE7										
1	0	1.22	1.64	27	++	+	-	+	26	2.6
2	1	0.4	0.32	6	nd	nd	nd	nd	85	17.8
3	2	0.15	0.33	4	+	nd	nd	nd	115	21.5
4	4	0.11	0.27	4	-	nd	nd	nd	nd	nd
pSLE1										
1	0	0.89	0.91	8	+	-	+	-	98	14.7
2	1	0.56	0.56	6	+	nd	nd	nd	107	16.7
3	5	0.12	0.34	0	nd	nd	nd	nd	120	23.3
4	13	0.11	0.45	0	+	nd	nd	nd	nd	nd
pSLE2										
1	0	0.78	0.45	6	+	-	+	-	82	3.0
2	1	0.45	0.33	2	nd	nd	nd	nd	100	6.5
3	7	0.34	0.24	2	+	nd	nd	nd	99	9.0
4	13	0.45	0.20	2	-	nd	nd	nd	nd	nd
pSLE24										
1	0	0.55	1.01	4	+	-	+	-	86	0
2	6	0.45	0.89	4	nd	nd	nd	nd	132	6.4
3	12	0.34	0.66	0	+	nd	nd	nd	nd	nd
4	64	0.45	0.70	0	+	nd	nd	nd		22.6

nd=not determined

Supplementary Table S8 (continued): Clinical and diagnostic parameters of pSLE patients in longitudinal study.

Patient/visit nr	Time after onset (months)	a-D5	a-CTD	SLEDAI	ANA	Sm	APL	a-RNP	C3	C4
pSLE3										
1	0	1.4	1.3	25	+	+	+	+	14	7
2	3	1.8	1.1	30	+	nd	+	nd	33	6
3	14	1.2	1.4	22	nd	nd	nd	nd	45	8
4	64	1.7	0.7	28	nd	nd	nd	nd	80	11
pSLE5										
1	0	1.1	1.3	24	+	-	-	+	22	1.9
2	3	1.6	0.78	26	nd	nd	nd	nd	55	4.4
3	6	1.45	0.65	22	+	nd	nd	nd	63	22
4	12	1.11	0.77	20	+	nd	nd	nd	79	54
pSLE8										
1	0	1.4	0.9	25	+	+	-	+	18	2.7
2	3	0.8	0.7	4	+	nd	nd	+	62	8.7
3	6	1.1	0.4	18	nd	nd	-	nd	35	3.6
4	18	0.6	0.2	6	-	nd	-	nd	104	15
pSLE9										
1	0	1.5	0.9	30	+	+	-	+	25	5
2	3	1.6	0.56	13	+	nd	nd	nd	85	22
3	6	0.77	0.3	8	nd	nd	nd	nd	nd	44
4	12	0.8	0.5	8	+	nd	-	+	140	nd

nd=not determined

S6. Detailed protocols for molecular dynamics

The binding of three modifications of the DNA 21-mer to the monoclonal antibody ED-10 (PDB ID: 2OK0) (5) were studied using the classical molecular dynamics (MD) approach.

Each simulated system was first energy-minimized, then heated to 310 K. The simulation protocol was similar to the one employed in earlier studies (6-8), however, slightly different in the three cases After heating, simulated system (i) was first equilibrated for 10 ns with harmonic restraints applied to the protein, and the dTdC basepair fixed in space as in the crystal structure. Next, the dTdC basepair was released, while the protein was still harmonically restrained, and the system was simulated for further 10 ns. Finally, all atoms were allowed to move and further 12.5 ns of simulations were performed under NPT ensemble conditions and using Nos´e-Andersen Langevin piston pressure control[5-7], allowing the systems to acquire a constant volume at 1 atm pressure. After equilibration, a 100 ns MD simulation was carried out in the NVT ensemble that was used for analysis. The root mean square displacement (RMSD) calculated for all atoms of the antibody proteins showed that the performed equilibration was sufficient to ensure a stable antibody structure.

In the case of systems (ii) and (iii), the pre-equilibrated structure of system (i) was used for the dC→dA (system ii) and dC→dT (system iii) mutations. The obtained mutants were then further equilibrated for 3 ns each, before a 100 ns production run for each system was carried out. The molecular mutations and structure analysis was performed with VMD (9).

Supplementary Figure S8: Equilibration of the dsDNA-antibody complex in MD simulations.*

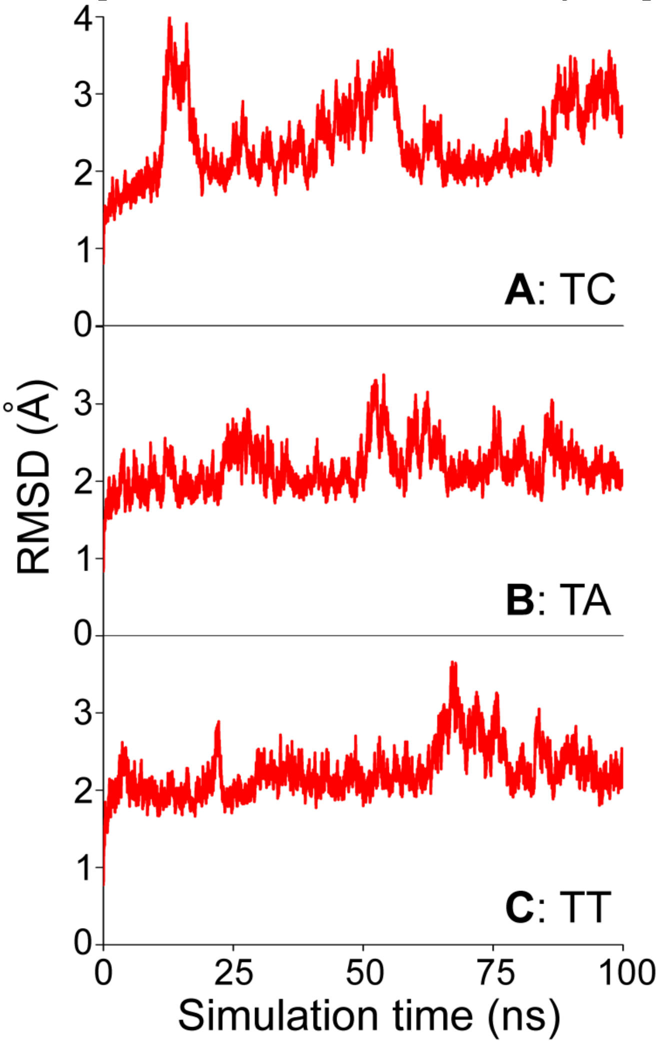

* Root-mean-square-displacement (RMSD) values calculated for all atoms of the antibody proteins in the dsDNA-antibody complex linked through the dCdT base pair (**A**), dAdT base pair (**B**), and dTdT base pair (**C**). All RMSD plots were computed with respect to the reference structure obtained after initial pre-equilibration, as described in Methods.

Figure S9: Analysis of dsDNA hydrogen bonding network.*

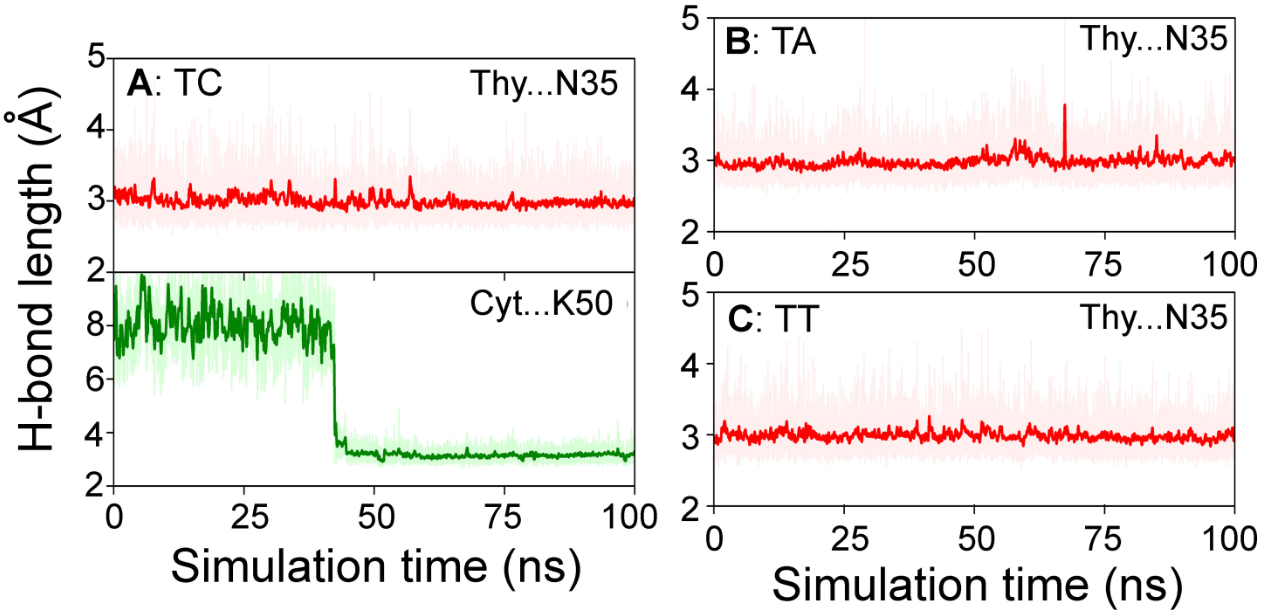

* Time evolution of the key hydrogen bond lengths stabilizing dsDNA binding to the antibody. For the dsDNA bonded through the dCdT base pair (**A**), there are two important hydrogen bonds that each nucleotide forms (these bonds are indicated with dashed lines in Fig. 5B), while only one significant hydrogen bond could be resolved over an interval of 100 ns for the dAdT base pair (**B**), and dTdT base pair (**C**). The hydrogen bond length is defined as the distance between heavier atoms (O and N), as indicated in Fig. 5B.

Figure S10: Stacking interactions in dsDNA-antibody binding.*

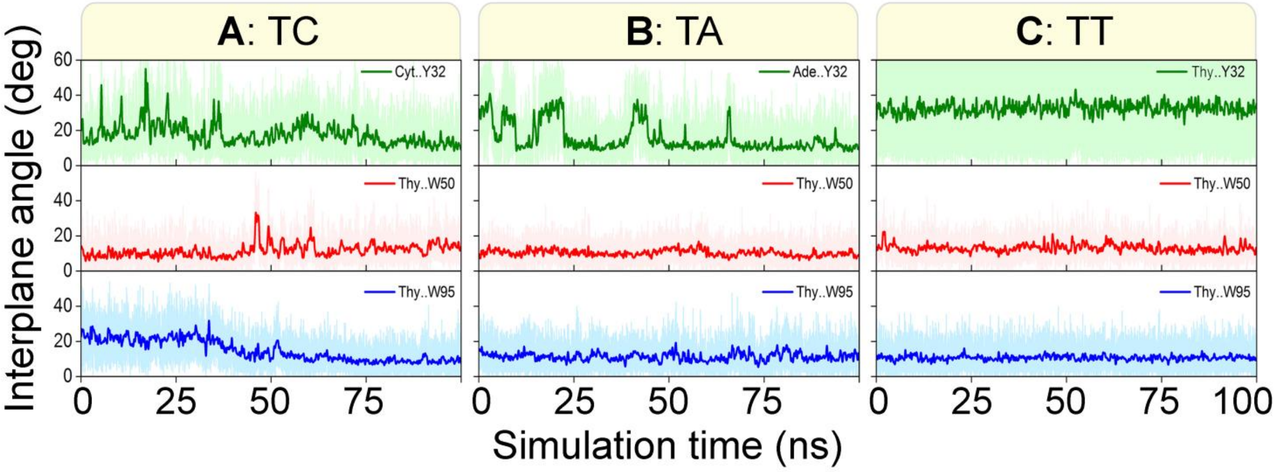

* Time evolution of the interplane angles computed between side chains of nucleotides from the dCdT base pair (**A**), dAdT base pair (**B**), and dTdT base pair (**C**) and the Y32 (green), W50 (red), and W95 (blue) residues side chains from the antibody (see Fig. 5B for the definition of the stacked pairs). Simulations indicate that these side chains are stacking above each other, and, therefore, the deviation of the interplane angle from zero is a measure for weakening of the stacking interactions, which is partially responsible for holding dsDNA and the antibody together. The angles calculated for each step of the simulation are shown with the shaded colors, while intense colors show the values averaged over 50 MD-steps.

Figure S11: dsDNA interaction with the antibody.*

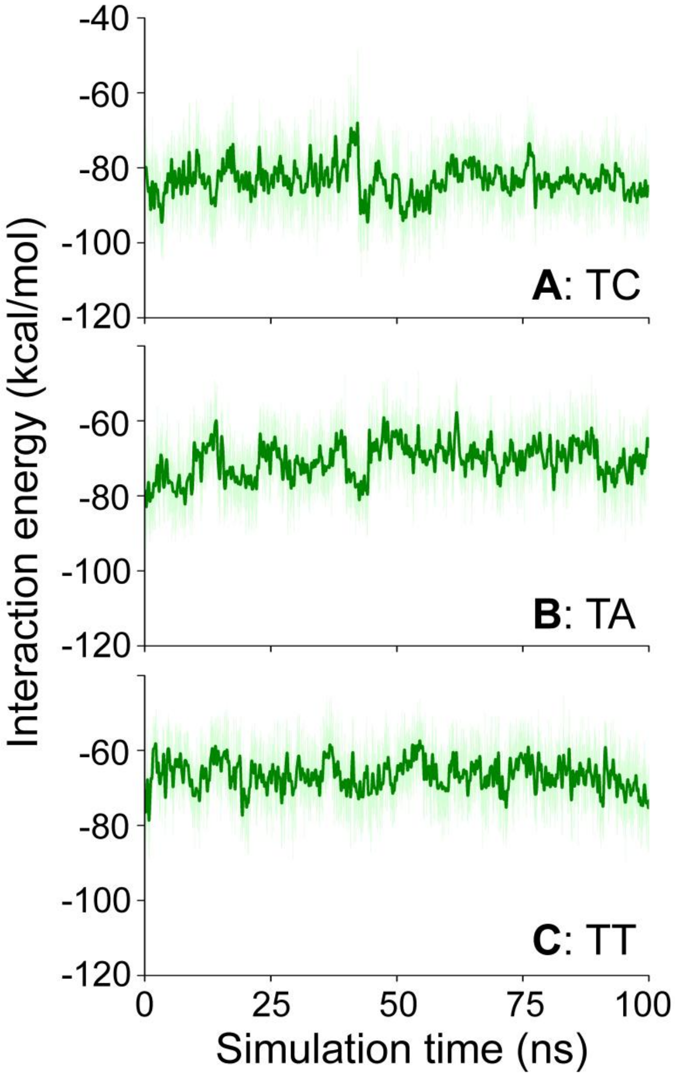

* Shown is the time evolution of the interaction energy for the dCdT base pair (**A**), dAdT base pair (**B**), and dTdT base pair (**C**) from the dsDNA and the antibody, attached to it. The energies calculated for each step of the simulation are shown with the shaded colors, while intense colors show energies averaged over 50 MD-steps.

References

1. Altman, D.G. Practical Statistics for Medical Research. London, 1991.

2. Maxwell, S. E. & Delaney, H. D. Designing Experiments and Analyzing Data: A Model Comparison Perspective. Second edition, New York, 2003.

3. Zar, J. H. Biostatistical Analysis. Fifth edition, Prentice Hall, NJ, 2010.

4. Cupples, L. A., Heeren, T., Schatzkin, T. A. & Colton, T. Multiple testing of hypotheses in comparing two groups. Ann Intern Med. 1984, 100, 122-129.

5. Sanguineti, S., Crowley, J. C., Merlo, M. L., Cerutti, M., Wilson, I., Goldbaum, F., Stanfield, R., & de Prat-Gay, G. *J. Mol. Biol.*, 370, 183–195 (2007).

6. Humphrey, W., Dalke, A. & Schulten, K. *J. Molec. Graphics*, 14, 33–38 (1996).

7. MacKerell Jr., A. D., Feig, M. & Brooks III, C. L. *J. Comp. Chem.*, 25, 1400–1415 (2004).

8. Barragan, A.M., Crofts, A. R., Schulten, K, & Solov'yov, I.A., *J. Phys. Chem. B*, 119, 433–447 (2015).

9. Solov'yov, I.A., Domratcheva, T., Moughal Shahi, A.R., & Schulten, K. *J. Am. Chem. Soc.*, 134, 18046–18052 (2012).
